# Neurorehabilitation including Virtual-Reality-Based Balance Therapy: Factors Associated with Training Response

**DOI:** 10.3390/brainsci14030263

**Published:** 2024-03-07

**Authors:** Evelyne Wiskerke, Jan Kool, Roger Hilfiker, Martin Sattelmayer, Geert Verheyden

**Affiliations:** 1 Department of Rehabilitation Sciences, KU Leuven—University of Leuven, 3001 Leuven, Belgium; 2 Rehazentrum Valens, Kliniken Valens, 7317 Valens, Switzerland; 3 Physiotherapy Tschopp & Hilfiker, 3902 Glis, Switzerland; 4 School of Health Sciences, HES-SO Valais-Wallis, 3954 Leukerbad, Switzerland

**Keywords:** digital therapeutics, virtual reality, exergaming, balance, stroke, multiple sclerosis, neurorehabilitation, therapy response

## Abstract

Background: Virtual reality (VR) therapy is increasingly used and has shown encouraging effects. Yet, it is unknown which patients respond best to VR-based balance therapy as part of neurorehabilitation. Methods: Data from 30 persons with stroke and 51 persons with multiple sclerosis who performed three to four weeks of VR-based balance therapy during in-patient rehabilitation were analysed. Participants were divided into responders and nonresponders based on achievement of the minimal clinically important difference in functional balance post intervention. Measures of balance, trunk function, mobility, gait, motivation, and exergame parameters were compared between groups. Results: Post intervention, all clinical measurements significantly improved (*p* < 0.05; effect size: 0.45–0.59). Participants that achieved the minimal clinically important difference in functional balance (n = 49; 60%) had significantly lower preintervention functional and dynamic balance (median(IQR): 39(27–46) versus 45(37–50); *p* = 0.02 and 11(6–15) versus 16(11–18); *p* = 0.03). They spent less time on higher difficulty exercises (11(8–17) versus 14.5(10–12); *p* = 0.03) and demonstrated increased motivation over time compared with nonresponders (1(−1–5) versus −2(−7–3); *p* = 0.03). Conclusion: Lower baseline balance ability, spending more time on adequately challenging exercises, and increased motivation potentially influence response to therapy. These factors can support the personalisation of VR-based balance therapy.

## 1. Introduction

Balance impairments are common in various neurological populations, such as in persons with stroke (PwS) and persons with multiple sclerosis (PwMS), affecting approximately 50% of individuals [1,2]. This leads to reduced gait performance, loss of independence in activities of daily living, recurrent falls, and the negative consequences thereof [3]. Therefore, improvement of balance is one of the main objectives during neurorehabilitation.

Balance can be trained through many different therapy modalities, e.g., in a functional-exercise-based manner or utilising various rehabilitation technologies. Virtual reality (VR) therapy is gaining popularity and being increasingly used during neurorehabilitation. Virtual reality therapy includes exercises performed in a virtual environment with a therapeutic focus, such as improving muscle strength, general flexibility, or balance ability, in which physical movements are needed to perform a specific activity or complete a task [4]. VR-based therapy offers an enjoyable, engaging, and challenging environment, potentially enhancing treatment effects in PwS and PwMS [5,6]. It is an effective training modality in addition to conventional therapy to improve balance and gait ability [7,8,9].

Within previous work by this research group [10], a VR exergame hierarchy was determined to optimally target VR balance exergames to the specific balance abilities of PwS and PwMS. Selecting the most appropriate exergame for the user ensures that the user is neither under- nor overchallenged, promotes continuous motivation during treatment, and potentially facilitates greater therapeutic results. In addition, understanding factors associated with response to therapy is important to optimise therapy effectiveness further. Knowledge of these factors can support allocating specific forms of therapy to a specific subset of patients.

Various prediction models have shown in the stroke population which initial factors influence long-term outcomes, i.e., measurements of sitting balance and lower extremity function early post stroke can predict independent gait three to six months post stroke [11,12]. Furthermore, previous research into factors that influence rehabilitation outcomes has identified the impact of initial motor impairment severity, chronicity, and cognitive function on the efficacy of neurorehabilitation [13,14,15]. While this addresses the first question, “What is this patient’s potential for recovery?”, posed by the authors from the Stroke Recovery and Rehabilitation Roundtable, it does not address the second question: “What is the best rehabilitation strategy for this person given her/his clinical profile?” [16]. This is where personalised rehabilitation is receiving increased interest, which involves exploring specific patient characteristics that influence treatment efficacy to allow for developing personalised rehabilitation programs with improved outcomes. Research in the domain of cancer therapy has made significant steps, whereby specific treatments for specific forms of cancer are known to have the highest potential treatment effect [17]. However, within neurorehabilitation, evidence is still scarce in this respect. The investigation of factors associated with responses to a specific form of therapy, such as balance and trunk therapy, is a relatively new area of research and has been explored in only a few studies.

Prat-Luri et al. [18] identified through meta-analysis various effect modifiers that influence the effectiveness of trunk training programs. Studies that included participants with higher initial trunk impairment resulted in a higher impact of treatment on trunk function and balance ability. Additionally, studies with older participants had a greater effect on trunk function, limits of stability, and functional mobility but surprisingly not on balance ability. Schwenk et al. [19] found similar results for sensor-based VR balance training. They uncovered through linear regression that low baseline balance ability was associated with greater improvement in balance abilities after training as measured by the centre of mass sway area. Werner et al. [20] identified early responders, i.e., persons that achieve a steep improvement curve within the first weeks of therapy, with exercise-based balance training among persons with dementia. Univariate analysis showed that lower baseline exergame performance and lower divided attention and visuospatial abilities predicted an early response to therapy. Surprisingly, even though motivation is seen as an important determinant of rehabilitation outcomes and is often described as having a positive impact [21], the studies mentioned earlier did not include the assessment of motivation.

In summary, very few studies have investigated factors influencing the response to VR-based balance therapy in PwS and PwMS. Nevertheless, acquiring knowledge about factors associated with therapy responses to this specific form of therapy can support its application, potentially optimising its effectiveness within both the clinical setting and research studies. Therefore, the purpose of the present study was (1) to investigate the change from preintervention to post intervention in PwS and PwMS during in-patient rehabilitation, including VR-based balance therapy; (2) to assess the relationship between clinical measurements of balance, trunk function, mobility and gait, and VR-based balance therapy; and (3) to explore the characteristics of the subgroups of responders and nonresponders to VR-based balance therapy.

## 2. Methods

This study is a secondary analysis of data collected from a longitudinal observational single-centre study. PwS and PwMS underwent three to four weeks of VR-based balance therapy during in-patient rehabilitation [10]. Ethical approval for this study was obtained from the Ethics Committee of the Sudostschweiz, BASEC number 2018-01248 (according to the declaration of Helsinki [22]). Each participant was adequately informed about the study, offered their collaboration, and signed a written informed consent.

### 2.1. Participants

Data from 30 PwS and 51 PwMS from the original dataset were used in this analysis. The following criteria were utilised for inclusion: (1) persons suffering from a recent stroke or multiple sclerosis (MS) with an Expanded Disability Status Scale between 3 and 6.5 (i.e., patients with moderate to severe disability who need assistance while walking), (2) aged over 18, (3) referred for a minimum of three weeks in-patient rehabilitation, (4) with reduced balance as measured by a Berg Balance Scale score of fewer than 52 out of 56 points, and (5) signed informed consent. Persons were excluded from the study if they had comorbidities that interfered with VR-based balance therapy performance, walking, or balance training (e.g., visual or cognitive impairments, psychiatric disorders, musculoskeletal problems). Standard care consisted of various therapies, including physical therapy, occupational therapy, aquatic therapy, robotic-assisted therapy, speech therapy, neuropsychology, and other additional therapy activities. Depending on the type of therapy, therapy was performed one-on-one or in a group setting.

### 2.2. Device and Therapy Program

**Figure 1:**
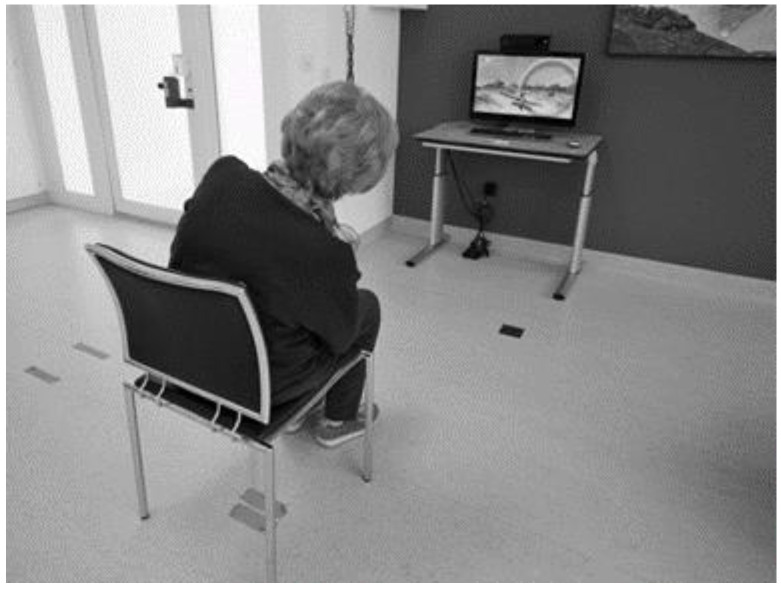
A participant performing therapy with the device in a seated position. A lateroflexion of the trunk to the right bodyside steers the aeroplane through the target, i.e., the circle. Source: Wiskerke et al. [10] available via https://games.jmir.org/2022/1/e30366/, accessed on 12 January 2024. This is an open-access article distributed under the terms of the Creative Commons Attribution License (https://creativecommons.org/licenses/by/4.0/, accessed on 12 January 2024), which permits unrestricted use, distribution, and reproduction in any medium.

**Figure 2:**
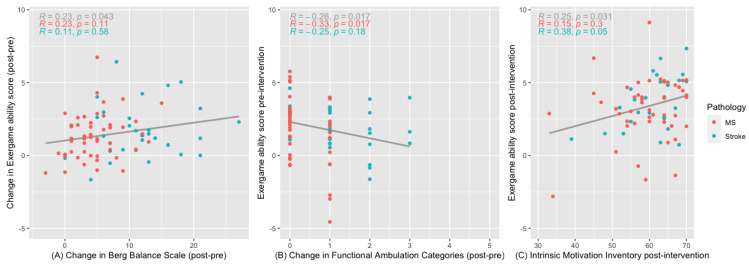
Significant correlations between clinical outcome measurements and exergame ability score.

Participants performed VR-based balance therapy with the MindMotion^TM^ GO device (MindMaze, Lausanne, Switzerland), as shown in Figure 1. Body movements were tracked by a Microsoft Kinect camera version 2 and were translated by the software into avatar movements to execute the exergame. Nine different exergames that targeted balance training in sitting and standing were available. More information regarding the different exergames can be found in Wiskerke et al.—Figure 2 [10].

PwS performed, on average, ten therapy sessions over four weeks, whereas PwMS performed, on average, seven therapy sessions within three weeks. Within one session, the participant performed six exergames with a total duration of twelve minutes. To ensure variety, different exergames were played during each therapy session. An assistant guided the therapy under the supervision of a trained physical therapist who specialised in technology-assisted therapy.

### 2.3. Collected VR Exergame Parameters

Various parameters from the performed VR exergames were collected, including the name of the exergame, the difficulty level, the position of execution (i.e., sitting or standing), and the total duration in minutes. Moreover, participants rated the subjective difficulty level of the exercises on a six-point Likert scale, where zero indicates the exercise is very easy, and five indicates it is impossible for the user to execute. The average subjective difficulty level is the average difficulty of all exergames performed in the first and last session. In addition, we investigated the number of minutes the participants performed exergames with too high difficulty. The objective difficulty summarised the minutes the participant spent on exergames in which they achieved a game score below 66%, meaning the exercises were too challenging. Subjective difficulty includes the play duration of exercises in which participants subjectively scored the exercise as challenging (i.e., a score of 2). The device score (in %, ranging from 0% to 100%) resulting from the VR exergames was used to perform a Rasch analysis with a repeated measure design [23]. From this analysis, the person’s ability in logits was calculated for each training session for each participant, henceforth called the exergame ability score.

### 2.4. Clinical Outcome Measurements

Demographic data and clinical measurements of trunk function, balance, gait, mobility, fatigue, and cognition were collected preintervention. The complete test battery of clinical measurements was repeated post intervention. Motivation was registered using a subset of the intrinsic motivation inventory directly after the first and last exergame therapy session.

#### 2.4.1. Berg Balance Scale

Basic balance was assessed with the Berg Balance Scale, a sensitive and specific measurement in the neurological population [24,25]. The scale consists of fourteen items, with scores ranging from 0 to 4 points and a maximum score of 56 points. Test–retest, interrater, and intrarater reliability were confirmed [24].

#### 2.4.2. Trunk Impairment Scale

The Trunk Impairment Scale assessed static and dynamic sitting balance and trunk coordination. The scale consists of seventeen items with a total score of 23 points, meaning normal trunk function. Sufficient reliability and internal validity of the scale were shown in the stroke and MS population [26,27].

#### 2.4.3. Dynamic Gait Index

Dynamic balance and gait were assessed with the Dynamic Gait Index, which is an eight-item scale scored on an ordinal scale with a total score of 24 points. It is a recommended scale in the subacute stroke population, as well as in the in-patient rehabilitation of PwMS with an EDSS up to 5.5 [28,29,30,31]. It was shown that the test can discriminate well between fallers and nonfallers in PwMS [28]. The dynamic gait index has good test–retest and interrater reliability in the stroke and MS population [29,30,31].

#### 2.4.4. Timed Up and Go Test

The Timed Up and Go Test was performed to evaluate walking ability, general mobility, and dynamic balance in a relevant daily life task. This test measures a participant’s time to stand up from a chair, walk 3 m, turn, return to the chair, and sit down. The test shows good construct validity with the Berg Balance Scale and good test–retest reliability in the stroke population [31]. Within the MS population, good convergent validity was found between the Timed Up and Go Test and other outcome measures of mobility, disability status, and balance confidence [32].

#### 2.4.5. Functional Ambulation Categories

The Functional Ambulation Categories (FACs) were used to classify the patients into one of six categories that describe if and how the patient ambulates and how much support from another person is needed for weight-bearing and maintaining balance. The test has excellent test–retest and interrater reliability in the stroke population [33]. Also, an excellent relationship exists between the FACs and the gait velocity and cadence within the stroke and MS population [34].

#### 2.4.6. Intrinsic Motivation Inventory

The intrinsic motivation inventory (IMI) is a multidimensional measurement including seven subscales to assess participants’ subjective experience of a specific activity [35]. Within the IMI, the items are stable across various tasks, conditions, and settings. As seen in other studies, multiple subscales can be selected as deemed appropriate for the purpose of the study [36,37,38,39]. For this study, the participants rated ten questions from the combined subscales of interest, enjoyment, and effort after the first and last VR balance therapy session.

### 2.5. Data Analysis

Initially, demographic data were analysed descriptively, and all clinical outcome measurements were assessed for normality using the Shapiro–Wilk test. Depending on the test for normality, the change in clinical outcome measurements and exergame ability score was assessed with the dependent *t*-test or Wilcoxon signed-rank test. The effect size was calculated with product r, with small, medium, and large effect sizes being r = 0.1, r = 0.3, and r = 0.5, respectively [40]. In the second step, the relationship between the clinical measurements and exergame ability score was assessed using either a Pearson correlation coefficient or Spearman’s Rank correlation coefficient, respectively [41].

Lastly, participants were divided into two groups based on their pathology-specific minimal clinically important difference (MCID) on the Berg Balance Scale, i.e., 2.5 points for PwMS [42] and 12.5 points for PwS [43]. Participants improving equally to, or more than, the minimal clinically important difference were classified into the group of responders, whereas participants improving below the MCID were classified as nonresponders. Then, demographics, clinical outcome measurements at baseline, change in motivation, and various exergame parameters of the exercises were compared between groups using independent *t*-tests or the Mann–Whitney U test, depending on the sample distribution assessed at baseline.

## 3. Results

### 3.1. Overview

Demographics are presented in Table 1, with the count and percentages or the median and interquartile range. In total, 81 participants were included in the study, of which 30 were PwS and 51 were PwMS. The median age was 57, with an interquartile range from 51 to 66 years. Slightly more women participated in the study than men (57% and 43%, respectively). The participants presented a low-to-moderate balance ability with a median Berg Balance Scale of 42 points. As demonstrated by the FAC, 84% of participants could ambulate without manual support.

### 3.2. Change over Time

First, the progression over time was investigated. Boxplots are presented in Table 2 with the median, interquartile range, the *p*-value of the Wilcoxon signed-rank test, and their respective effect size (product–moment r). A significant improvement was seen for exergame ability, Berg Balance Scale, Trunk Impairment Scale, Dynamic Gait Index, and Functional Ambulation Categories but not for the Intrinsic Motivation Inventory throughout neurorehabilitation. The effect size for significant results was moderate to large, ranging from 0.45 to 0.58. A separate analysis for PwS and PwMS can be found in Appendix A and Appendix B.

### 3.3. Correlation between Clinical Outcome Measurements and Exergame Ability Score

The correlation between the various clinical outcome measurements and the exergame ability score was explored through Spearman correlation coefficients (Appendix C). Correlations between baseline and postintervention, as well as change over time, were explored. The BBS change over time showed a mild significant positive correlation with the exergame ability change over time (Figure 2A). The FAC change preintervention was mildly significantly correlated with the exergame ability at baseline (Figure 2B). The IMI postintervention was mildly correlated with the exergame ability postintervention (Figure 2C). No other significant correlations were found (see Appendix C for all correlations).

### 3.4. Comparison of Responders to Nonresponders

A comparison of responders with nonresponders is presented in Table 3. The responder group consisted of relatively more PwMS. In comparison, the nonresponder group included relatively more PwS (*p* = 0.004). Balance activity as measured by the Berg Balance Scale and the Dynamic Gait Index was significantly lower preintervention in the responder group (BBS median 39 and 45 points, respectively, *p* = 0.015; DGI median 11 and 16 points, respectively, *p* = 0.030). Regarding the exergame parameters, it was revealed that the nonresponder group performed significantly more exercises that were objectively too difficult, i.e., participants achieved a score below 66% (*p* = 0.034). However, subjective difficulty was viewed similarly in both groups. Interestingly, motivation at baseline was similar in both groups. Still, there was a significant difference between groups in change in motivation, whereby the nonresponder group displayed a reduction in motivation. In contrast, the responder group saw an increase in motivation (median −2 and 1 point, respectively, *p* = 0.032). There was no statistical difference between groups regarding gender, weakest bodyside, cognition, trunk function, gait ability, mobility at baseline, exergame score at baseline, or number of sessions performed in total.

## 4. Discussion

In line with previous research [7,8,9], a significant positive change over time was observed in balance ability, trunk function, gait, and exergame ability during in-patient rehabilitation incorporating VR-based balance therapy. Furthermore, weak, significant positive correlations were found between changes in exergame ability and both balance and gait ability. Motivation showed a weak significant positive correlation with the exergame ability post intervention. Further investigation into the factors associated with the response to VR-based balance therapy revealed that participants categorised as responders had a significantly lower baseline balance ability. Participants categorised as nonresponders performed significantly more exercises that were of excessive difficulty. Most interestingly, even though motivation at baseline was comparable between groups, the responder group displayed an increase in motivation over time. In contrast, the nonresponder group showed a reduction in motivation.

An improvement in the clinical outcome measurements for balance, trunk function, gait, and mobility was expected after neurorehabilitation treatment and is in line with the current literature reporting on the effectiveness of neurorehabilitation and specific forms of VR-based balance therapy [6,7,8,9]. However, motivation did not show significant changes over time. While VR exercises tend to elicit a positive initial reaction with moderate-to-high motivation scores, studies examining the development of motivation during rehabilitation find that this level of motivation often remains stable or occasionally decreases [39,44,45].

Unexpectedly, we found predominantly weak significant correlations between the exergame ability and clinical outcome measurements (including motivation). One reason for this could be that the VR exergame ability is based on many different exergames with various aims, levels of difficulty, playing positions (i.e., sitting or standing), and movement components (i.e., static versus dynamic balance). All these components are integrated into the model, providing the exergame ability, thus increasing the variation and potentially explaining the low correlations.

Forty-nine participants (60%) were classified as responders and thirty-two (40%) as nonresponders, a similar division to Werner et al. [20]. It was noticed that the responder group included more persons with MS. The MCID used in our study of PwMS was three times lower than the one utilised in PwS. The advantage of using pathology-specific MCIDs is the adaptation to the pathology. However, one of the MCIDs could potentially be more suitable to the setting of the study, thereby potentially leading to an under- or overclassification of responders. Furthermore, responders displayed lower balance ability, both in static and dynamic balance ability preintervention. This is in line with previous results that show initial impairment to influence outcomes after rehabilitation [13,15,18]. Moreover, Schwenk et al. [19] demonstrated that lower baseline balance performance was associated with a greater improvement in balance outcomes after VR therapy. Noteworthily, exergame ability at baseline was not significantly different between groups.

A closer look at the exergame parameters reveals that participants in the nonresponder group performed more exercises where they achieved low scores. Still, the subjective difficulty score was similar between groups. Notably, participants in both groups experienced the exercises overall as equally difficult. Moreover, motivation decreased in the nonresponder group but increased in the responder group throughout the intervention. Motivation is a concept frequently used by therapists and is considered an important determinant of rehabilitation outcomes [21]. As shown by Widmer et al. [44], positive feedback provided through game parameters and rewards supports motivation during VR-based therapy and potentially supports motor skill improvements. Motivation depends on the level of challenge, feedback delivery, state of immersion, and various other exercise parameters [39,46]. It could be hypothesised that the settings of the game parameters were too difficult and, in reaction, led to a reduction in motivation. When performing exercises that are too challenging, participants receive reduced positive feedback and reward, potentially leading to a lower response to therapy. Lastly, the total therapy duration was not significantly different between groups. However, the nonresponders received a median of 12 more minutes of therapy and experienced a more considerable variation in the total duration of therapy. Therapy doses are viewed as being important for the improvement of rehabilitation outcomes, as it is commonly claimed that more training leads to better results [47]. However, specifically for balance exercises, dosage depends not only on the time spent training but also on the difficulty of the exercise and the balance ability of the person, which can vary from day to day [48], thus requiring consideration for therapeutic measures.

As this was an explorative study, a secondary analysis of data from a longitudinal observational study was used to explore factors associated with therapy response to VR-based balance therapy in PwS and PwMS. Various learnings from this study should be considered when reviewing the results and designing potential predictive models. The MCID for PwMS for the Berg Balance Scale is similar within the literature, ranging between three to four points [42,49]. There is a broader range in MCID in PwS reported in the literature, ranging from 4 to 12.5 points [43,50,51]. This can be explained by the variation in the study setting, the phase of stroke, and the statistical method used to define the MCID. In the current study, we opted for the MCID of 12.5 points, as the study by Song et al. [43] showed the most similarity with our clinical setting and phase poststroke. Nevertheless, using such a high MCID could have potentially influenced our results, as it remains a single cut-off point with its inherent disadvantages [52].

To avoid dichotomising participants into two groups, a multilinear regression could have been used. In a post hoc analysis, we explored how the exergame ability changed over time and extracted the coefficient of improvement through linear regression as a more stable and less influenceable value of progress. However, correlations between the coefficient of improvement and the various clinical outcome measurements were weak (rho < 0.2). Thus, the analysis could not be used to produce reliable results. Future studies should focus their study design primarily on collecting data to define predictive factors of VR-based balance therapy to allow for more continuous and robust statistical methods.

The personalisation of rehabilitation is highly clinically relevant. Positive improvements are apparent after rehabilitation, including VR-based therapy. However, differences in improvement are potentially influenced by baseline performance, the difficulty of VR-based exercises, and the motivation throughout rehabilitation. Future research should focus the study design primarily on acquiring knowledge on these and other factors associated with therapy response to support the development of personalised therapy regimes and optimising their effectiveness within neurorehabilitation.

## 5. Conclusions

A significant improvement in balance ability, trunk function, gait, and exergame ability was found after in-patient rehabilitation, including VR-based balance therapy. Moreover, exergame ability demonstrated weak correlations with clinical outcome measurements of balance, gait ability, and motivation. Investigation of participants that responded positively to VR-based balance therapy, indicated by an improvement on the Berg Balance Scale, revealed that a lower baseline ability, spending more time on adequately challenging exercises, and less time on too difficult exercises resulting in low scores and an increase in motivation potentially contribute to a positive therapy response. In summary, this study provides new insights into factors associated with the response to VR-based balance therapy, which can facilitate the development of a therapy regime that best matches the patient’s profile, abilities, and needs. In the future, these findings should be verified in a larger study with a primary focus on developing a model to facilitate personalised rehabilitation.

## Figures and Tables

**Table 1:** Demographics.

Characteristic	All (n = 81)	Stroke (n = 30)	MS (n = 51)
**Age, (years) median (IQR)**	57 (51–66)	65 (56–77)	55 (46–60)
**Gender, n (%)**			
Male	35 (43)	22 (73)	13 (25)
Female	46 (57)	8 (27)	38 (75)
**Stroke, n (%)**	30 (37)		
Ischemic		27 (90)	n.a.
Haemorrhagic		3 (10)	n.a.
**Multiple sclerosis, n (%)**	51 (63)		
Primary-progressive MS		n.a.	13 (25)
Secondary-progressive MS		n.a.	19 (37)
Relapse-remitting MS		n.a.	19 (37)
**Hemiparetic or weaker body side, n (%)**			
Left	38 (47)	12 (40)	26 (51)
Right	40 (49)	16 (53)	24 (47)
Bilateral	3 (4)	2 (7)	1 (2)
**Time post diagnosis, median (IQR)**			
Time post stroke (days)		14 (11–21)	n.a.
Time since MS diagnosis (years)		n.a.	16 (10–21)
**Montreal Cognitive Assessment score, median (IQR)** _[range 0–30]_	24 (22–26)	23 (20–25)	25 (23–27)
**Exergame ability score, median (IQR)**	2.18 (0.83–3.07)	1.64 (0.83–3.19)	2.34 (1.24–3.02)
**Berg Balance Scale score, median (IQR)** _[range 0–56]_	42 (29–47)	41 (26–47)	44 (34–47)
**Trunk Impairment Scale score, median (IQR)** _[range 0–23]_	17 (14–18)	16 (13–19)	17 (15–18)
**Dynamic Gait Index score, median (IQR)** _[range 0–24]_	13 (8–17)	11 (0–17)	13 (8–17)
**Timed Up and Go test time (seconds), median (IQR)**	18 (11–31)	21 (12–33)	16 (11–28)
With walking aid, n (%)	38 (47)	19 (63)	35 (69)
Without walking aid, n (%)	43 (53)	11 (37)	16 (31)
**Functional Ambulation Category, n (%)**			
0–2	13 (16)	13 (43)	0 (0)
3–5	68 (84)	17 (57)	51 (100)
**Intrinsic Motivation Inventory, median (IQR)** _[range 0–70]_	61 (54–64)	63 (60–66)	60 (54–64)

Footnote: results are presented in a count (n) and a respective % of the total number of participants. Furthermore, the median and interquartile range (IQR) are presented.

**Table 2:** Change over time of clinical measurements.

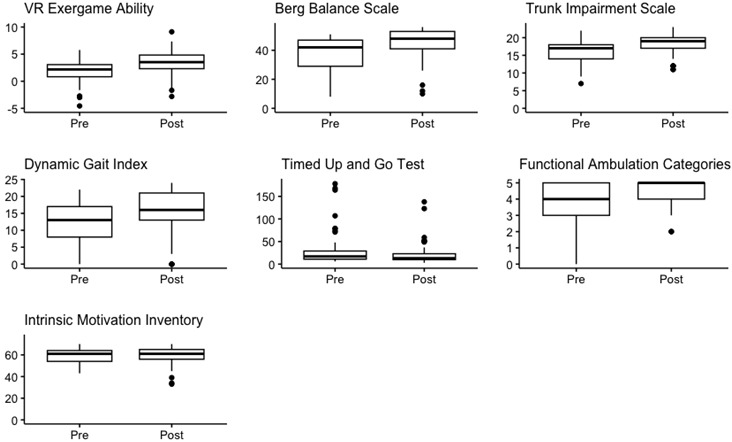				
	Preintervention Median (IQR)	Postintervention Median (IQR)	*p*-Value	Effect Size
**VR Exergame ability**	2.18 (0.83–3.07)	3.53 (2.32–4.83)	<0.001	0.50
**Berg Balance Scale** _[range 0–56]_	42 (29–47)	48 (41–53)	<0.001	0.59
**Trunk Impairment Scale**_[range 0–23]_	17 (14–18)	19 (17–20)	<0.001	0.50
**Dynamic Gait Index** _[range 0–24]_	13 (8–17)	16 (13–21)	<0.001	0.52
**Timed Up and Go Test**	18 (11–31)	13 (10–23)	<0.001	0.55
**Functional Ambulation Categories** _[range 0–5]_	4 (3–5)	5 (4–5)	<0.001	0.45
**Intrinsic Motivation Inventory** _[range 0–70]_	61 (54–64)	61 (56–65)	0.54	0.05

Footnote: Pre- and postintervention median scores with their interquartile range are presented for each outcome, as well as the respective *p*-value of the Wilcoxon signed-rank test with effect size (r).

**Table 3:** Comparison of responder with nonresponder group.

Variable	Nonresponder (n = 32)	Responder (n = 49)	*p*-Value
Demographic variables			
Age	62 (54–73)	56 (51–61)	0.13
Gender	18 female;14 male	28 female; 21 male	0.94 **
Diagnosis	18 stroke; 14 MS	12 stroke; 37 MS	0.00 *
Weaker bodyside	2 bilateral; 12 left; 16 right	1 bilateral; 26 left; 24 right	0.18 **
Cognitive function (MoCA)	24 (21–26)	25 (22–27)	0.34
Clinical measures at baseline			
Berg Balance Scale score t0 _[range 0–56]_	45 (37–50)	39 (27–46)	0.02
Trunk Impairment Scale score t0 _[range 0–23]_	17 (15–19)	16 (13–18)	0.13
Dynamic Gait Index score t0 _[range 0–24]_	16 (11–18)	11 (6–15)	0.03
Timed Up and Go test time t0	14 (11–26)	21 (13–33)	0.17
Functional Ambulation Category t0 _[range 0–5]_	4 (3–5)	4 (3–5)	0.54
Intrinsic motivation t0 _[range 0–70]_	61 (54–64)	61 (54–64)	0.84
Development of motivation over time			
Intrinsic motivation change _[range 0–70]_	−2 (−7–3)	1 (−1–5)	0.03
Exergame parameters			
Exergame ability score baseline, logits	2.02 (0.735–3.015)	2.23 (1.26–3.190)	0.50
Total duration, minutes	96 (84–120)	84 (84–98)	0.13
Subjective average difficulty _[range 0–5]_	1.64 (1.46–1.93)	1.65 (1.45–1.98)	0.99
Exercises with objective high difficulty, n	15 (10–21)	11 (8–17)	0.03
Exercises with subjective high difficulty, n	26 (20–36)	29 (19–35)	0.78

Footnote: * significant results with *p* < 0.05; ** Chi-squared test was used.

## Data Availability

The datasets used and/or analysed during the current study are available from the corresponding author upon reasonable request.

## Appendix A. Change over Time of Clinical Measurements in Persons with Stroke

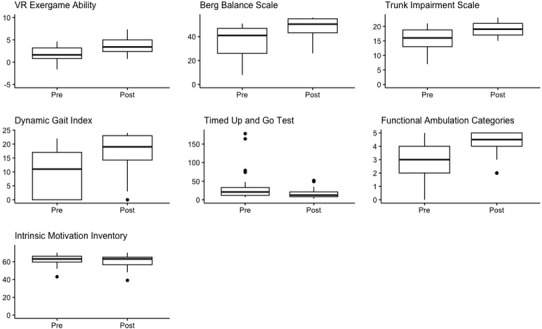

	**Preintervention** **Median (IQR)**	**Postintervention** **Median (IQR)**	***p*** **-Value**	**Effect Size**
**VR Exergame ability**	1.64 (0.83–3.19)	3.41 (2.38–5.00)	<0.001	0.52
**Berg Balance Scale** _[range 0–56]_	41 (26–47)	51 (43–55)	<0001	0.61
**Trunk Impairment Scale** _[range 0–23]_	16 (13–19)	19 (17–21)	<0.001	0.58
**Dynamic Gait Index** _[range 0–24]_	11 (0–17)	19 (14–23)	<0.001	0.61
**Timed Up and Go Test**	16 (11–28)	13 (10–24)	<0.001	0.68
**Functional Ambulation Categories** _[range 0–5]_	3 (2–4)	5 (4–5)	<0.001	0.60
**Intrinsic Motivation Inventory** _[range 0–70]_	63 (60–66)	63 (57–65)	0.54	0.08

## Appendix B. Change over Time of Clinical Measurements in Persons with Multiple Sclerosis

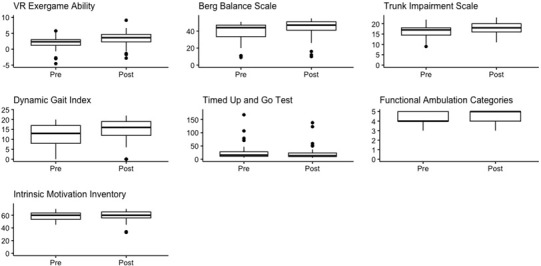

	**Preintervention** **Median (IQR)**	**Postintervention** **Median (IQR)**	***p*** **-Value**	**Effect Size**
**VR Exergame ability**	2.34 (1.24–3.02)	3.63 (2.31–4.68)	<0.001	0.49
**Berg Balance Scale** _[range 0–56]_	44 (34–47)	47 (41–51)	<0.001	0.57
**Trunk Impairment Scale** _[range 0–23]_	17 (15–18)	18 (16–20)	<0.001	0.42
**Dynamic Gait Index** _[range 0–24]_	13 (8–17)	16 (12–19)	<0.001	0.43
**Timed Up and Go Test**	16 (11–28)	13 (10–24)	<0.001	0.50
**Functional Ambulation Categories** _[range 0–5]_	4 (4–5)	5 (4–5)	<0.001	0.36
**Intrinsic Motivation Inventory** _[range 0–70]_	60 (54–64)	60 (56–65)	0.28	0.10

## Appendix C. Spearman Rho Correlations between Clinical Measurements and Exergame Ability Score

Footnote: PreInt, represents the preintervention measurement; PostInt, the postintervention measurement; Delta, the difference between pre–post measurements; BBS, Berg Balance Scale; TIS, Trunk Impairment Scale; FAC, Functional Ambulation Categories. * Significant correlation, *p* < 0.05.
